# Prognostic and predictive value of YTHDF1 and YTHDF2 and their correlation with tumor-infiltrating immune cells in non-small cell carcinoma

**DOI:** 10.3389/fonc.2022.996634

**Published:** 2022-11-21

**Authors:** Young Wha Koh, Jae-Ho Han, Seokjin Haam, Hyun Woo Lee

**Affiliations:** ^1^ Department of Pathology, Ajou University School of Medicine, Suwon, South Korea; ^2^ Department of Thoracic and Cardiovascular Surgery, Ajou University School of Medicine, Suwon, South Korea; ^3^ Department of Hematology-Oncology, Ajou University School of Medicine, Suwon, South Korea

**Keywords:** non-small cell lung cancer, YTHDF1, YTHDF2, CD8, CD4, FOXP3

## Abstract

**Background:**

YTH domain-containing family protein 1 (YTHDF1) or YTHDF2 play crucial roles in cancer immunotherapy. We examine the expression of YTHDF1, YTHDF2, CD8, CD4, and FOXP3 to identify their prognostic or predictive role for PD-1/PD-L1 inhibitor in non-small cell lung cancer (NSCLC).

**Methods:**

Immunohistochemical expression of YTHDF1, YTHDF2, CD8, CD4, and FOXP3 was investigated in 266 patients not receiving PD-1/PD-L1 inhibitors and in 59 patients receiving PD-1/PD-L1 inhibitors. Immunohistochemical results were verified using mRNA dataset obtained from The Cancer Genome Atlas (TCGA) database.

**Results:**

Immunohistochemical expression of YTHDF1 or YTHDF2 was negatively associated with CD8- and CD4-positive T cells; however, the same expression was positively associated with FOXP3-positive T cells. YTHDF1 or YTHDF2 mRNA expression was also negatively associated with CD8- and CD4-positive T cells. Gene set enrichment analysis revealed that low YTHDF1 was related to immune hot tumor gene sets. Expression of YTHDF1 or YTHDF2 was negatively associated with expression of most immune checkpoints. YTHDF1 and YTHDF2 were predictive markers of response to PD-1/PD-L1 inhibitors. YTHDF1 or YTHDF2 expression was associated with better prognosis. YTHDF1 has an immune hot profile in both cell types, whereas YTHDF2 is only seen in adenocarcinoma.

**Conclusion:**

Low YTHDF1 or YTHDF2 reflects an immune hot tumor signature and may serve as a predictor or prognostic marker.

## Introduction

Anti-programmed cell death protein 1/programmed death-ligand 1 (PD-1/PD-L1) drugs have been approved for treatment of patients with advanced non-small cell lung cancer (NSCLC) (1–3). The expression of PD-L1 by tumor cells has been focused on as the best marker of sensitivity to PD-1/PD-L1 inhibitors (4). However, durable response to anti-PD-1/PD-L1 inhibitor have also been reported in PD-L1-negative patients (5). Various predictors, including tumor mutational burden (6), tumor-infiltrating lymphocytes (7), and immune-related gene signatures (8), are also candidate biomarkers; however, these biomarkers have not been validated. Furthermore, in the clinic, the evaluation of tumor mutational burden or immune-related gene signatures is difficult because it requires expensive techniques, including next-generation sequencing or nanostring technology.

N6-methyl adenosine (m^6^A), is responsible for post-transcriptional modification of mRNA in most eukaryotes (9). The m^6^A pathway components play important roles in oncogene-mediated cell transformation (10), cell proliferation and tumorigenicity (11, 12), and tumor progression (13). The YTH domain-containing family protein 1 (YTHDF1), a component of the m^6^A pathway, affects mRNA translation efficiency (14). Recently, Han et al. reported an important effect of YTHDF1 in the antitumor immunity (15). In melanoma and colon cancer models, YTHDF1 knockout mice showed favorable outcomes and increased CD8 positive T cells and NK cells (15). Furthermore, in a melanoma cancer model, the frequency of tumor regression to anti-PD-L1-treatment was increased in YTHDF1 knockout mice than in wild-type mice (15). YTHDF2 induces NSCLC growth by enhancing mRNA translation of 6-phosphogluconate dehydrogenase (16). YTHDF2 also promotes tumor proliferation by increasing CDKN1B mRNA degradation in intrahepatic cholangiocarcinoma (17). YTHDF2 expression was negatively associated with PD-L1 in esophageal cancer (18). Tsuchiya et al. revealed that YTHDF1 or YTHDF2 expression showed better clinical outcomes in NSCLC (19). Previous findings suggest that YTHDF1 or YTHDF2 may be a therapeutic target for cancer immunotherapy or a predictive biomarker predicting the response to anti-PD-1/PD-L1. However, there are no studies on the predictive role of YTHDF1 or YTHDF2 in NSCLC patients receiving PD-1/PD-L1 inhibitor. Although the role of YTHDF1 or YTHDF2 in the tumor immune microenvironment may differ depending on cell type, no such study has been performed.

Our study investigated the prognostic significance of YTHDF1 or YTHDF2 expression in a cohort of 266 patients who did not receive PD-1/PD-L1 inhibitor. We further investigated whether expression of YTHDF1 or YTHDF2 affected the response in a group of 59 patients treated with PD-1/PD-L1 inhibitor. Correlation analyses of YTHDF1, YTHDF2, and tumor infiltrating lymphocytes (CD4- and CD8-positive T cells and FOP3-positive T regulatory cells (Treg)) were performed on immunohistochemical and gene expression data. We also performed gene set enrichment analysis (GSEA) using The Cancer Genome Atlas (TCGA) to identify overexpressed gene classes based on YTHDF1 or YTHDF2 expression. In order to identify the tumor immune microenvironment associated with the expression of YTHDF1 or YTHDF2, the association between such expression and immune checkpoints other than PD-1/PD-L1 was investigated. All experiments were performed in two cell types (adenocarcinoma and squamous cell carcinoma).

## Materials and methods

### Study population and patient characteristics

Our study included a cohort of 266 patients not receiving PD-1/PD-L1 inhibitors and a group of 59 patients receiving PD-1/PD-L1 inhibitors. PD-1/PD-L1 inhibitor blockade was used in all patients from 2016 to 2022 and their drug responses were evaluated. The ethical approval was approved by the Institutional Review Board of Ajou University School of Medicine (AJIRB-BMR-KSP-19-416 and 2019-11-11). Complete response, partial response, or stable disease was defined as the responder group, and disease progression was defined as the non-responder group (20). Patient characteristics are summarized (**Table 1**). In the group not receiving PD-1/PD-L1 inhibitor treatment, 64% had adenocarcinoma and 29% had advanced stage. In the group treated with PD-1/PD-L1 inhibitor, 61% had adenocarcinoma and all were advanced stage. All patients receiving PD-1/PD-L1 inhibitor were previously refractory to chemotherapy, radiation therapy, or targeted agents. Twenty-five patients (42.4%) were responders

**Table 1:** Demographic and clinical characteristics of patients.

Variable	Non-treatment group of PD-1/PD-L1 Inhibitors (n = 266)	Treatment group of PD-1/PD-L1 Inhibitors (n = 59)
Age, median (range) (years)	63 (31–86)	67 (32–81)
Male sex	188 (70.7%)	51 (86.4%)
Smoking history	167 (67.3%)	35 (83.3%)
Histologic subtype		
Adenocarcinoma	171 (64.3%)	36 (61%)
Squamous cell carcinoma	95 (35.7%)615	20 (33.9%)
Not otherwise specified	0 (0%)	3 (5.1%)
pTNM 8th edition		
Unclassified	7 (2.6%)	0 (0%)
Stage I	107 (40.2%)	0 (0%)
Stage II	74 (27.8%)	0 (0%)
Stage III	78 (29.3%)	18 (30.5%)
Stage IV	0 (0%)	41 (69.5%)
Type of PD-1 blockade		
Nivolumab	–	23 (39%)
Pembrolizumab	–	13 (22%)
Atezolizumab		23 (39%)
Response to PD-1 blockade		
Responder	–	25 (42.4%)
Non-responder	–	34 (57.6%)

### Immunohistochemistry of YTHDF1, YTHDF2, CD8, CD4 and FOXP3

Antibodies YTHDF1 (polyclonal, Proteintech), YTHDF2 (polyclonal, Proteintech), CD8 (clone C8/144B, DAKO), CD4 (clone SP35, Cell Marque), and FOXP3 (clone 236A/E7, Abcam) were used. The intensity of YTHDF1 or YTHDF2 staining was defined in four categories: 0, 1, 2, 3. The percentages of cytoplasmic or membranous expression were also evaluated. H-scores were applied to examine the YTHDF1 or YTHDF2 stains (21). For interpretation of CD4, CD8, or FOXP3 cells, membrane-positive CD4 or CD8 cells or nuclear-positive FOXP3 cells were measured at three locations at 400x magnification in the tumor area and averaged.

### Gene expression analysis

mRNA data of 1018 NSCLCs (517 lung adenocarcinoma and 501 squamous cell carcinoma) obtained from TCGA cBioportal were used. (http://cbioportal.org) (22).

GSEA is a method to analyze underlying biological processes using mRNA expression. We performed GSEA using GSEA version 4.0.3 (23). We analyzed data based on the median value of YTHDF1 or YTHDF2 expression. The Hallmark gene set was used as the gene set database. If *p* < 0.05 and false discovery rate (FDR) < 0.25, it was defined as statistically significant. Web-based Kaplan Meier plotter tool was used for survival analyses (24). Survival analysis was performed using mRNA data from 719 adenocarcinomas and 524 squamous cell carcinomas.

### Statistical analyses

Correlation between quantitative variables was determined using Spearman’s method. Logistic regression analysis was performed to identify predictive biomarker for anti-PD-1/PD-L1. The cutoffs of YTHDF1 and YTHDF2 were determined using receiver operating curve (ROC) analysis. Kaplan–Meier estimator was used for survival analysis. A cox proportional hazard model was used for survival multivariate analysis. IBM SPSS Statistics for Windows (Version 25.0. Armonk, NY, USA) was used, and a *p*-value less than 0.05 was defined as statistically significant.

## Results

### Correlation among YTHDF1, YTHDF2, CD4, CD8, and FOXP3 analyzed by mRNA expression and immunohistochemistry

**Figure 1:**
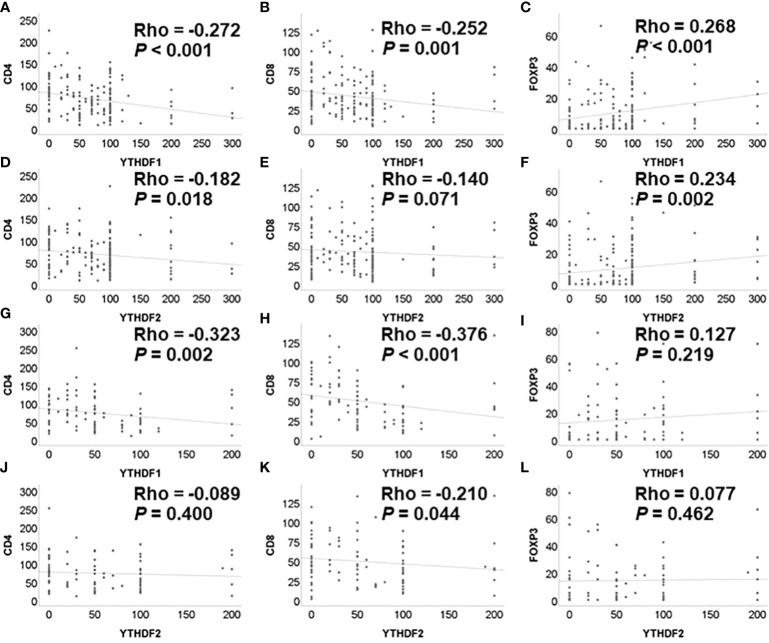
Correlation among YTHDF1, YTHDF2, CD4, CD8, and FOXP3 analyzed by immunohistochemistry in PD-1 inhibitor non-treatment groups. **(A)** YTHDF1 and CD4 in adenocarcinoma. **(B)** YTHDF1 and CD8 in adenocarcinoma. **(C)** YTHDF1 and FOXP3 in adenocarcinoma. **(D)** YTHDF2 and CD4 in adenocarcinoma. **(E)** YTHDF2 and CD8 in adenocarcinoma. **(F)** YTHDF2 and FOXP3 in adenocarcinoma. **(G)** YTHDF1 and CD4 in squamous cell carcinoma. **(H)** YTHDF1 and CD8 in squamous cell carcinoma. **(I)** YTHDF1 and FOXP3 in squamous cell carcinoma. **(J)** YTHDF2 and CD4 in squamous cell carcinoma. **(K)** YTHDF2 and CD8 in squamous cell carcinoma. **(L)** YTHDF2 and FOXP3 in squamous cell carcinoma.

The correlation analysis of YTHDF1, YTHDF2 and tumor infiltrating lymphocytes in the non-treatment group is summarized in **Figure 1** In the adenocarcinoma group not receiving PD-1/PD-L1 inhibitor, the immunohistochemical expression of YTHDF1 was significantly negatively associated with CD4 and CD8 and positively correlated with FOXP3 expression. The immunohistochemical expression of YTHDF2 was also significantly negatively associated with CD4 and positively correlated with FOXP3 expression. In the squamous cell carcinoma group not receiving PD-1/PD-L1 inhibitor, the immunohistochemical expression of YTHDF1 was significantly negatively associated with CD4 and CD8 expression. The immunohistochemical expression of YTHDF2 was also significantly negatively associated with CD8 expression.

In the adenocarcinoma group receiving PD-1/PD-L1 inhibitor treatment, the immunohistochemical expression of YTHDF1 was significantly negatively associated with CD4 expression (**Supplementary Figure 1**).

**Figure 2:**
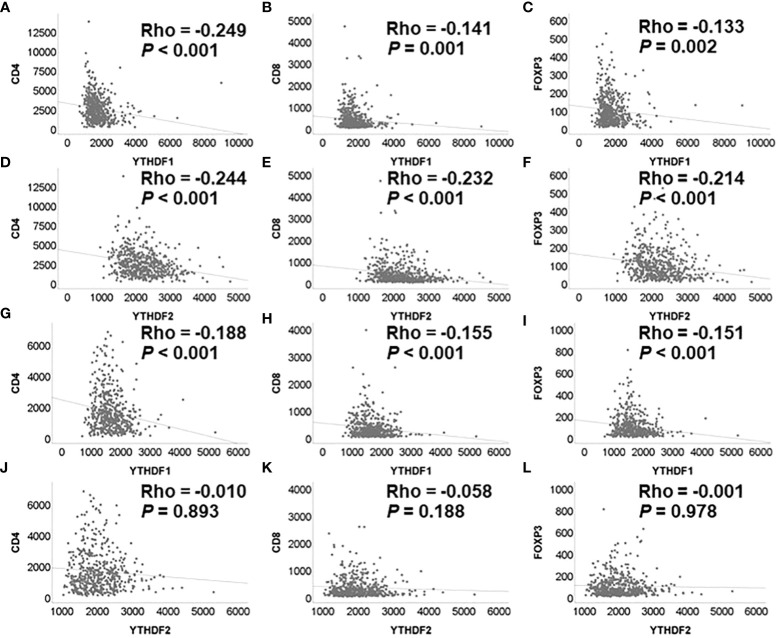
Correlation among YTHDF1, YTHDF2, CD4, CD8, and FOXP3 analyzed by mRNA expression in PD-1 inhibitor non-treatment groups. **(A)** YTHDF1 and CD4 in adenocarcinoma. **(B)** YTHDF1 and CD8 in adenocarcinoma. **(C)** YTHDF1 and FOXP3 in adenocarcinoma. **(D)** YTHDF2 and CD4 in adenocarcinoma. **(E)** YTHDF2 and CD8 in adenocarcinoma. **(F)** YTHDF2 and FOXP3 in adenocarcinoma. **(G)** YTHDF1 and CD4 in squamous cell carcinoma. **(H)** YTHDF1 and CD8 in squamous cell carcinoma. **(I)** YTHDF1 and FOXP3 in squamous cell carcinoma. **(J)** YTHDF2 and CD4 in squamous cell carcinoma. **(K)** YTHDF2 and CD8 in squamous cell carcinoma. **(L)** YTHDF2 and FOXP3 in squamous cell carcinoma.

**Figure 3:**
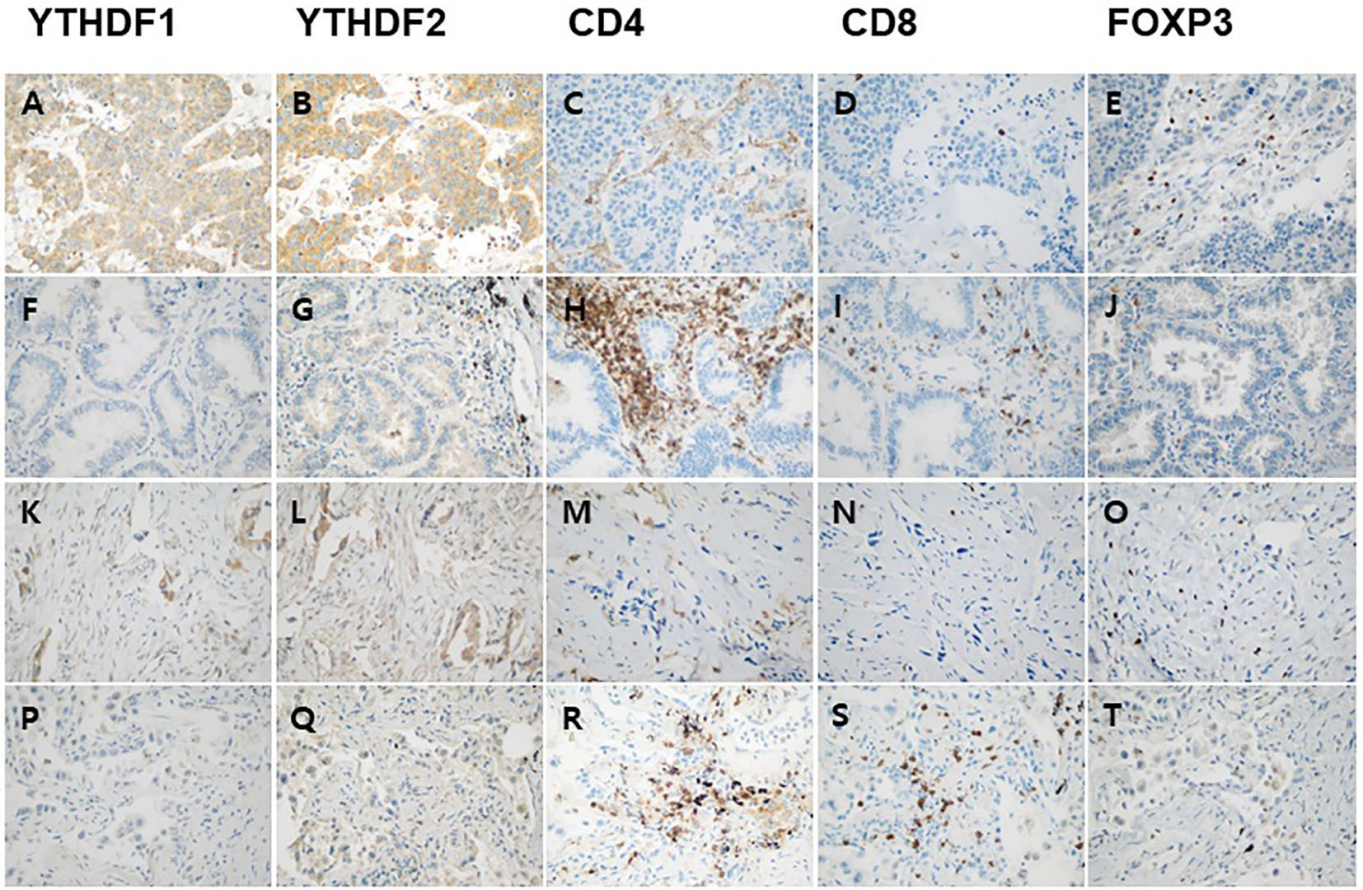
Representative immunohistochemical images of YTHDF1, YTHDF2, CD4, CD8, and FOXP3 expression in adenocarcinoma. High YTHDF1 **(A)** or YTHDF2 **(B)** case is associated with low CD4 **(C)**, CD8 **(D)** and high FOXP3 **(E)** expression in PD-1 inhibitor non-treatment group. Low YTHDF1 **(F)** or YTHDF2 **(G)** case is associated with high CD4 **(H)**, CD8 **(I)** and low FOXP3 **(J)** expression in PD-1 inhibitor non-treatment group. High YTHDF1 **(K)** or YTHDF2 **(L)** case is associated with low CD4 **(M)**, CD8 **(N)** and high FOXP3 **(O)** expression in PD-1 inhibitor treatment group. Low YTHDF1 **(P)** or YTHDF2 **(Q)** case is associated with high CD4 **(R)**, CD8 **(S)** and low FOXP3 **(T)** expression in PD-1 inhibitor treatment group.

Correlation analyses among YTHDF1, YTHDF2, and tumor infiltrating lymphocytes were performed using the mRNA expression data of TCGA. The YTHDF1 or YTHDF2 mRNA expression was significantly negatively associated with CD4, CD8, and FOXP3 mRNA expression in adenocarcinoma (**Figure 2**). The YTHDF1 mRNA expression was also significantly negatively associated with CD4, CD8, and FOXP3 mRNA expression in squamous cell carcinoma (**Figure 2**). The YTHDF2 mRNA expression was not associated with CD4, CD8, and FOXP3 mRNA expression in squamous cell carcinoma (**Figure 2**). The YTHDF2 mRNA expression was not associated with CD4, CD8, and FOXP3 mRNA expression in squamous cell carcinoma (**Figure 2**). Representative figures of immunohistochemistry in adenocarcinoma show that high YTHDF1 or YTHDF2 cases are associated with low CD4, CD8 and high FOXP3 expression in both PD-1 inhibitor treatment and non-treatment groups (**Figure 3**). However, low YTHDF1 or YTHDF2 cases are associated with high CD4, CD8 and low FOXP3 expression in both PD-1 inhibitor treatment and non-treatment groups (**Figure 3**).

### Prognostic or predictive role of YTHDF1 or YTHDF2

In the adenocarcinoma group not receiving PD-1/PD-L1 inhibitor, the cutoff values of YTHDF1 and YTHDF2 were 60 and 75, respectively. In the squamous cell carcinoma group not receiving PD-1/PD-L1 inhibitor, the cutoff values of YTHDF1 and YTHDF2 were 45 and 40, respectively. Because the sample size of the PD-1/PD-L1 inhibitor-treated group was small, all cell types were combined for survival analysis. In the group receiving PD-1/PD-L1 inhibitor treatment, the cutoff values of YTHDF1 and YTHDF2 were 30 and 20, respectively.

**Figure 4:**
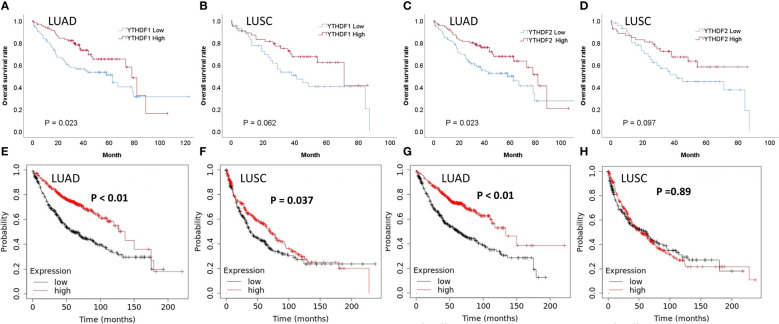
Survival analyses according to YTHDF1 and YTHDF2 expression in patients not receiving PD-1/PD-L1 inhibitors. **(A)** Overall survival (OS) and immunohistochemical expression of YTHDF1 in lung adenocarcinoma (LUAD). **(B)** OS and immunohistochemical expression of YTHDF1 in lung squamous cell carcinoma (LUSC). **(C)** OS and immunohistochemical expression of YTHDF2 in LUAD. **(D)** OS and immunohistochemical expression of YTHDF2 in LUSC. **(E)** OS and mRNA expression of YTHDF1 in LUAD. **(F)** OS and mRNA expression of YTHDF1 in LUSC. **(G)** OS and mRNA expression of YTHDF2 in LUAD. **(H)** OS and mRNA expression of YTHDF2 in LUSC.

In the adenocarcinoma group not receiving PD-1/PD-L1 inhibitor treatment, the immunohistochemical expression of YTHDF1 or YTHDF2 was correlated with better overall survival (*p* = 0.023, **Figure 4A** and *p* = 0.023, **Figure 4C**, respectively). In the squamous cell carcinoma group not receiving PD-1/PD-L1 inhibitor treatment, the immunohistochemical expression of YTHDF1 or YTHDF2 showed a trend toward better overall survival but was not statistically significant (*p* = 0.062, **Figure 4B** and *p* = 0.097, **Figure 4D**, respectively). In multivariate analysis, YTHDF1 or YTHDF2 immunohistochemical expression was an independent favorable prognostic marker for overall survival in adenocarcinoma patients (hazard ratio (HR) = 0.418, *p* = 0.001 and HR = 0.449, *p* = 0.001, respectively; **Table 2**). In Kaplan Meier plotter analysis, the group with high YTHDF1 mRNA expression showed better overall survival than the group with low YTHDF1 mRNA expression from adenocarcinoma or squamous cell carcinoma (*p* < 0.01, **Figure 4E** and *p* = 0.037, **Figure 4F**, respectively). The group with high YTHDF2 mRNA expression was also correlated with better overall survival in adenocarcinoma (*p* < 0.01, **Figure 4G**), although there was no difference in survival rate according to the level of YTHDF2 mRNA in squamous cell carcinoma (*p* = 0.89, **Figure 4H**).

**Table 2:** Univariate and multivariate analyses of overall survival in immunohistochemical data of non-treatment group of PD1/PDL1 Inhibitors.

Univariate analysis						
covariate	Adenocarcinoma			Squamous cell carcinoma		
	HR	95%CI	P-value†	HR	95%CI	*P*-value†
Age (≥65 y vs. <65 y)	1.326	0.828-2.125	0.240	1.217	0.764-1.939	0.409
Sex (male vs. female)	2.245	1.327-3.797	0.003	1.232	0.782-1.941	0.368
Stage (III–IV vs. I–II)	2.385	1.486-3.830	<0.001	2.665	1.701-4.178	<0.001
Smoking history (+ vs. -)	1.628	0.978-2.708	0.061	1.428	0.872-2.341	0.157
YTHDF1 (low vs. high)	0.583	0.364-0.934	0.025	0.704	0.434-1.141	0.154
YTHDF2 (low vs. high)	0.565	0.353-0.907	0.018	0.461	0.272-0.783	0.004
Multivariate analysis						
Covariate	Adenocarcinoma			Squamous cell carcinoma		
	HR	95%CI	P-value	HR	95%CI	*P*-value
Sex (male vs. female)	2.449	1.425-4.211	0.001	2.064	1.279-3.330	0.003
Stage (III–IV vs. I–II)	2.458	1.523-3.965	<0.001	2.064	1.279-3.330	0.003
YTHDF1 (low vs. high)	0.418	0.255-0.685	0.001	0.704	0.434-1.141	0.154
Sex (male vs. female)	2.132	1.248-3.642	0.006	2.064	1.279-3.330	0.003
Stage (III–IV vs. I–II)	2.330	1.445-3.755	0.001	2.064	1.279-3.330	0.003
YTHDF2 (low vs. high)	0.449	0.274-0.736	0.001	0.704	0.434-1.141	0.154

CI, confidence interval; HR, hazard ratio; LVI, lymphovascular invasion.

†cox proportional hazard model analysis.

We evaluated the predictive roles of YTHDF1, YTHDF2, and clinicopathologic variables on the response to PD-1/PD-L1 blockade. In univariate analysis, the group with low YTHDF1 expression was statistically more likely to respond to the PD-1/PD-L1 inhibitor than the group with high YTHDF1 expression (*p* = 0.003, **Table 3**). In multivariate analysis, the expression of YTHDF1 was an independent predictor for PD-1/PD-L1 blockade (*p* = 0.024, odd ratio (OR) = 0.189). Low expression of YTHDF2 was also statistically more likely to respond to PD-1/PD-L1 inhibitors in univariate analysis (*p* = 0.013, **Table 3**). In multivariate analysis, the expression of YTHDF2 was an independent predictor for PD-1/PD-L1 blockade (*p* = 0.031, OR = 0.196). We then performed survival analyses in the groups receiving PD-1/PD-L1 inhibitor treatment. The group with low YTHDF1 immunohistochemical expression had better progression-free survival and overall survival than the group with high YTHDF1 expression; however, there were not statistically significant (*p* = 0.154, **Supplementary Figure 2A** and *p* = 0.494, **Supplementary Figure 2B**, respectively). The immunohistochemical expression of YTHDF2 was also not correlated with progression-free survival or overall survival rate (*p* = 0.9, **Supplementary Figure 2C** and *p* = 0.967, **Supplementary Figure 2D**, respectively). We provided the immunhistochemical data of the PD-1 inhibitor treatment group and non-treatment group in the form of **Supplementary material Datasheet 1** (treatment group) and **Supplementary material Datasheet 2** (non-treatment group).

**Table 3:** Univariate and multivariate logistic regression analysis for predicting clinical response to PD-1/PD-L1 blockade.

Univariate analysis			
covariate	OR	95%CI	P-value†
Age (≥65 years vs.<65 years)	2.125	0.724-6.233	0.170
Sex (male vs. female)	6.222	0.713-54.29	0.098
Histology (ADC vs. non-ADC)	0.929	0.322-2.674	0.891
PD-L1 (≥50% vs. <50%)	3.030	0.991-9.268	0.052
YTHDF1 (high vs. low)	0.159	0.046-0.545	0.003
YTHDF2 (high vs. low)	0.187	0.050-0.700	0.013
Multivariate analysis			
Covariate	OR	95%CI	P-value†
Age (≥65 years vs.<65 years)	1.032	0.261-4.086	0.964
Sex (male vs. female)	9.066	0.769-106.8	0.080
PD-L1 (≥50% vs. <50%)	4.281	1.110-16.51	0.035
YTHDF1 (high vs. low)	0.189	0.045-0.805	0.024
Age (≥65 years vs.<65 years)	2.138	0.574-7.959	0.257
Sex (male vs. female)	5.784	0.526-63.61	0.151
PD-L1 (≥50% vs. <50%)	5.094	1.282-20.23	0.021
YTHDF2 (high vs. low)	0.196	0.045-0.865	0.031

ADC, adenocarcinoma; CI, confidence interval; OR, odd ratio; PD-L1, programmed Death-Ligand 1.

†Logistic regression analysis.

### GSEA and correlation analysis between YTHDF1, YTHDF2, and immune checkpoints

GSEA was performed using mRNA data obtained from TCGA. In lung adenocarcinoma, the low YTHDF1 group was mainly enriched in immunity-related signaling pathways (allograft rejection, IL6-JAK-STAT3 signaling, inflammatory response, and IL2-STAT5 signaling) (**Table 4**). In lung squamous cell carcinoma, the low YTHDF1 group was also mainly enriched in immunity-related signaling pathways (allograft rejection, IL2-STAT5 signaling, inflammatory response, IL6-JAK-STAT3 signaling, and TNFA signaling *via* NFKB and interferon gamma response). In lung adenocarcinoma, the low YTHDF2 group was mainly enriched in immunity-related signaling pathways (inflammatory response, IL6-JAK-STAT3 signaling, allograft rejection, and IL2-STAT5 signaling). In lung squamous cell carcinoma, there was no immune-related gene set related to YTHDF2.

**Table 4:** Immune-related gene sets in GSEA.

NAME	SIZE	ES	NES	Nominal p-val	FDR q-val	FWER p-val
Gene sets related to low YTHDF1 in adenocarcinoma patients						
HALLMARK_ALLOGRAFT_REJECTION	164	0.609	1.884	0.014	0.102	0.057
HALLMARK_IL6_JAK_STAT3_SIGNALING	74	0.556	1.823	0.018	0.051	0.105
HALLMARK_INFLAMMATORY_RESPONSE	172	0.493	1.727	0.022	0.089	0.203
HALLMARK_IL2_STAT5_SIGNALING	166	0.419	1.689	0.006	0.089	0.268
Gene sets related to low YTHDF1 in squamous cell carcinoma patients						
HALLMARK_ALLOGRAFT_REJECTION	164	0.705	2.172	0.000	0.000	0.000
HALLMARK_IL2_STAT5_SIGNALING	166	0.519	2.024	0.000	0.011	0.025
HALLMARK_INFLAMMATORY_RESPONSE	172	0.594	1.947	0.000	0.019	0.048
HALLMARK_IL6_JAK_STAT3_SIGNALING	74	0.633	1.939	0.000	0.017	0.051
HALLMARK_TNFA_SIGNALING_VIA_NFKB	166	0.551	1.813	0.018	0.033	0.137
HALLMARK_INTERFERON_GAMMA_RESPONSE	166	0.614	1.771	0.023	0.042	0.190
Gene sets related to low YTHDF2 in adenocarcinoma patients						
HALLMARK_INFLAMMATORY_RESPONSE	172	0.549	1.917	0.005	0.052	0.037
HALLMARK_IL6_JAK_STAT3_SIGNALING	74	0.558	1.803	0.021	0.084	0.139
HALLMARK_ALLOGRAFT_REJECTION	164	0.583	1.796	0.016	0.056	0.149
HALLMARK_IL2_STAT5_SIGNALING	166	0.430	1.733	0.002	0.057	0.213

ES, enrichment score; FDR, false discovery rate; FWER, family-wise error rate, NES, normalized enrichment score.

We then performed correlation analysis between YTHDF1, YTHDF2, and immune checkpoints using mRNA expression data. In lung adenocarcinoma, YTHDF1 is significantly negatively correlated with PD-L1, PD-1, PD-L2, CTLA-4, TIGIT, VISTA, and TIM3 (**Table 5**). In lung squamous cell carcinoma, YTHDF1 is significantly negatively correlated with PD-L1, PD-1, PD-L2, CTLA-4, TIGIT, VISTA, and TIM3. YTHDF2 is significantly negatively correlated with PD-L1, PD-1, PD-L2, CTLA-4, TIGIT, LAG3, VISTA, and TIM3 in lung adenocarcinoma. In lung squamous cell carcinoma, YTHDF2 is significantly negatively associated with PD-L1 and PD-L2.

**Table 5:** Correlations between YTHDF1, YTHDF2 and immune checkpoints in mRNA expression data.

	Adenocarcinoma (n = 517)				Squamous cell carcinoma (n = 501)			
	YTHDF1†	*p*	YTHDF2†	*p*	YTHDF1†	*p*	YTHDF2†	*p*
PD-L1	-0.087	0.048	-0.243	<0.001	-0.115	0.009	-0.268	<0.001
PD-1	-0.090	0.039	-0.302	<0.001	-0.118	0.007	-0.067	0.134
PD-L2	-0.194	0.001	-0.291	<0.001	-0.213	<0.001	-0.205	<0.001
CTLA-4	-0.130	0.002	-0.257	<0.001	-0.154	<0.001	-0.076	0.088
TIGIT	-0.130	0.003	-0.222	<0.001	-0.125	0.005	-0.016	0.720
LAG3	0.039	0.366	-0.242	<0.001	-0.055	0.217	-0.060	0.179
VISTA	-0.267	<0.001	-0.278	<0.001	-0.216	<0.001	-0.001	0.978
TIM3	-0.226	<0.001	-0.242	<0.001	-0.206	<0.001	-0.051	0.253

† Spearman’s correlation test

## Discussion

Protein expression of YTHDF1 or YTHDF2 was negatively correlated with CD8- and CD4-positive T cells, but positively correlated with Treg cells. The mRNA data also showed that the level of YTHDF1 was negatively correlated with CD8 and CD4 expression. In GSEA, low YTHDF1 mRNA expression was confirmed to be closely related to the immune-related pathway. The expression of YTHDF1 showed a negative correlation with most immune checkpoints. High YTHDF1 expression was associated with better prognosis. However, groups with low YTHDF1 or YTHDF2 expression were more likely to respond to PD-1/PD-L1 inhibitors than groups with high YTHDF1 expression. These results indicate that the low YTHDF1 and YTHDF2 groups are immune-inflamed tumors, also named “hot tumors.” Hot tumors are generally known to respond better to immunotherapy (25, 26). As expected, the low YTHDF1 and low YTHDF2 groups responded better to PD-1/PD-L1 inhibitor treatment. The expression of YTHDF1 or YTHDF2 in NSCLC can be a good predictive biomarker for PD-1/PD-L1 inhibitor.

m^6^A methylation plays important roles in regulating mRNA splicing, export, localization, translation, and stability (9). Only a few previous studies have reported on the relationship between YTHDF1 and cancer. Zhao et al. reported that YTHDF1 expression was associated with poor clinical outcomes in patients with hepatocellular carcinoma (27). Nishizawa et al. reported that the c-Myc oncogene promoted YTHDF1 expression and the knockdown of YTHDF1 resulted in the suppression of cell proliferation and sensitization to anticancer drugs in colorectal cancer (28). YTHDF2 is also a reader protein and plays an important role in regulating mRNA stability (29). High expression of YTHDF2 in ovarian cancer induces tumor progression (30). YTHDF2 is known to inhibit hepatocellular carcinoma cell proliferation and growth by inhibiting EGFR mRNA stability (31).

Han et al. reported that knockout of YTHDF1 resulted in higher levels of CD8+ T-cells and NK cells in melanoma and colon cancer mouse models (15). The knockout of YTHDF1 induced an increase in PD-L1 expression (15). In a melanoma cancer mouse model, tumor regression was found more frequently in anti-PD-L1-treated YTHDF1 knockout mice than in anti-PD-L1-treated wild-type mice (15). Our study also revealed that low expression of YTHDF1 was correlated with CD8 and CD4 protein or mRNA expression. Previous studies have shown that high CD4+ or CD8+ cells are associated with better responses to PD-1/PD-L1 blocking therapy. Before PD-1/PD-L1 blockade treatment, high level of peripheral blood CD4+ cells was associated with long-term survival (32). The transcriptome signature of PD-1 high CD8+ T cells showed a better prognosis in multiple cancers that underwent immune checkpoint inhibitor therapy (33). In our study, the expression of YTHDF1 was positively correlated with FOXP3. Treg cells are immunosuppressive and downregulate the induction and proliferation of effector T cells (34). Treg cells also play an important role in PD-1/PD-L1 therapy. Because Treg cells proliferate after PD-1/PD-L1 blockade, hyperprogression occurs during PD-1/PD-L1 blockade (35). Non-responders to PD-1/PD-L1 blocking therapy usually show an increase in PD-1 in Treg (36). The response was better when the ratio of tumor-infiltrating PD-1+CD8+T cells was higher than that of PD-1+Treg cells (36). The CD8 and CD4 high and FOXP3 low profile seen in the low YTHDF1 group indicates immune hot tumors and is a key factor in the response to PD-1/PD-L1 inhibitor.

In our study, YTHDF1 showed no difference in immune profile and prognosis according to cell type, although YTHDF2 showed a significant difference. In adenocarcinoma, YTHDF2 was negatively correlated with CD4 and CD8 and positively correlated with FOXP3 in protein and mRNA analysis. In squamous cell carcinoma, YTHDF2 showed a negative correlation with CD8 in protein analysis, but there were no correlations among YTHDF2, CD3, CD8, and FOXP3 in mRNA analysis. In GSEA of squamous cell carcinoma, there were no immune-related gene sets associated with YTHDF2. However, four immune-associated gene sets related to YTHDF2 were found in adenocarcinoma. In adenocarcinoma, all eight immune checkpoints showed a negative relationship with YTHDF2, but only two immune checkpoints were negatively correlated in squamous cell carcinoma. In Kaplan Meier plotter analysis, high YTHDF2 is associated with a better prognosis in adenocarcinoma, but YTHDF2 is not associated with prognosis in squamous cell carcinoma. Because YTHDF2 expression does not affect the immune profile of squamous cell carcinoma, there is no difference in survival rate.

In GSEA, the low YTHDF1 group was correlated with several immune-related pathways including IL2-STAT5 signaling, IL6-JAK-STAT3 signaling, and TNFA signaling *via* NFKB and interferon gamma response. The low YTHDF2 group was also associated with IL2-STAT5 and IL6-JAK-STAT3 signaling pathways. The association between immune-related pathways and PD-1/PD-L1 inhibitors has been reported several times in the past. IL2-STAT5 immune signatures are known to predict reactivity to PD-1/PD-L1 inhibitors (37). The IL-6/JAK1 pathway induces PD-L1 Y112 phosphorylation, leading to cancer immune evasion (38). TNF-α promotes PD-L1 expression in human prostate and colon cancer cells (39). The IFN-γ-related mRNA profile is a biomarker for PD-1 inhibitors that are currently attracting attention (40, 41).

High YTHDF1 or YTHDF2 expression was associated with better prognosis in immunohistochemistry and mRNA data sets. High YTHDF1 or YTHDF2 expression groups showed low immune checkpoint expression. Because immune checkpoints expressed on tumor cells protect tumor cells from attack by local immunity, the higher is the expression of immune checkpoints, the worse is the prognosis (42, 43). When treating with PD-1/PD-L1 inhibitor, the higher is the expression of immune checkpoints, the better is the expected response to treatment. In our low YTHDF1 or YTHDF2 expression group, immune checkpoint expression is high, indicating a good response to the PD-1/PD-L1 inhibitor. Similar to YTHDF1 and YTHDF2, PD-L1 expression is a poor prognostic factor in NSCLC (44, 45); however, the higher is the expression of PD-L1, the higher is the response rate to PD-1/PD-L1 inhibitor (4).

Our study had some limitations. First, ours was a retrospective observational study with a relatively small sample size. Second, we used an immunohistochemical method. However, immunohistochemistry has limitations regarding standardization, reliability, and reproducibility (46). Third, YTHDF1 or YTHDF2 expression was a predictive marker of response to PD-1/PD-L1 inhibitor but had no correlation with prognosis. Because the number of patients receiving PD-1/PD-L1 inhibitor was small (59 patients), our results need to be verified in a larger study. Forth, we performed immunohistochemical studies and mRNA studies on samples from different groups. Therefore, because protein or mRNA expression in the same sample is not compared, there is a limit to the analysis of protein and mRNA expression. Fifth, our study only confirmed the relationship between YTHDF1, YTHDF2, CD4, CD8, and FOXP3, however did not reveal which pathway YTHDF1, YTHDF2 affects on the tumor immune profile. Thereafter, experiments such as *in vivo* mouse models need to confirm our results and additional studies also determine how the YTHDF1 and YTHDF2 pathways affect immune profiles.

## Conclusion

Low YTHDF1 or YTHDF2 expression shows an immune hot profile of high CD8, high CD4, and low FOXP3. GSEA confirmed that low YTHDF1 or YTHDF2 tumor expression reflects the gene set of immune hot tumors. Low YTHDF1 or YTHDF2 showed higher expression of immune checkpoints than high YTHDF1 or YTHDF2. YTHDF1 or YTHDF2 was a predictive marker of response to PD-1/PD-L1 inhibitors. The expression of YTHDF1 or YTHDF2 was associated with prognosis. YTHDF1 has an immune hot profile in both lung adenocarcinoma and squamous cell carcinoma, whereas YTHDF2 is only seen in adenocarcinoma.

## Data availability statement

The original contributions presented in the study are included in the article/**Supplementary Material**. Further inquiries can be directed to the corresponding author.

## Ethics statement

The studies involving human participants were reviewed and approved by the Institutional Review Board of Ajou University School of Medicine (AJIRB-BMR-KSP-19-416 and 2019-11-11). The ethics committee waived the requirement of written informed consent for participation.

## Author contributions

Conception/design: YK. Provision of study material or patients: YK, J-HH, SH and HWL. Data analysis and interpretation: YK, J-HH, SH and HL. Manuscript writing: YK, J-HH, SH and HL. Final approval of manuscript: YK, J-HH, SH and HL. All authors contributed to the article and approved the submitted version.

## Funding

This research was supported by the Basic Science Research Program through the National Research Foundation of Korea (NRF) funded by the Ministry of Science, ICT (NRF-2020R1A2C1100568 for YK).

## Conflict of interest

The authors declare that the research was conducted in the absence of any commercial or financial relationships that could be construed as a potential conflict of interest.

## Publisher’s note

All claims expressed in this article are solely those of the authors and do not necessarily represent those of their affiliated organizations, or those of the publisher, the editors and the reviewers. Any product that may be evaluated in this article, or claim that may be made by its manufacturer, is not guaranteed or endorsed by the publisher.

## Supplementary material

Supplementary Figure 1 Correlation among YTHDF1, YTHDF2, CD4, CD8, and FOXP3 analyzed by immunohistochemistry in PD-1 inhibitor treatment groups. (A) YTHDF1 and CD4 in adenocarcinoma. (B) YTHDF1 and CD8 in adenocarcinoma. (C) YTHDF1 and FOXP3 in adenocarcinoma. (D) YTHDF2 and CD4 in adenocarcinoma. (E) YTHDF2 and CD8 in adenocarcinoma. (F) YTHDF2 and FOXP3 in adenocarcinoma. (G) YTHDF1 and CD4 in squamous cell carcinoma. (H) YTHDF1 and CD8 in squamous cell carcinoma. (I) YTHDF1 and FOXP3 in squamous cell carcinoma. (J) YTHDF2 and CD4 in squamous cell carcinoma. (K) YTHDF2 and CD8 in squamous cell carcinoma. (L) YTHDF2 and FOXP3 in squamous cell carcinoma.

Supplementary Figure 2 Survival analyses according to YTHDF1 and YTHDF2 expression in patients receiving PD-1/PD-L1 inhibitors. (A) Progression-free survival and expression of YTHDF1. (B) Overall survival and expression of YTHDF1. (C) Progression-free survival and expression of YTHDF2. (D) Overall survival and expression of YTHDF2.
