# Supplementary figures and images for: Prognostic and predictive value of YTHDF1 and YTHDF2 and their correlation with tumor-infiltrating immune cells in non-small cell carcinoma
Source: Front Oncol. 2022 Nov 21;12:996634. doi: 10.3389/fonc.2022.996634 (PMC9720116; doi:10.3389/fonc.2022.996634)

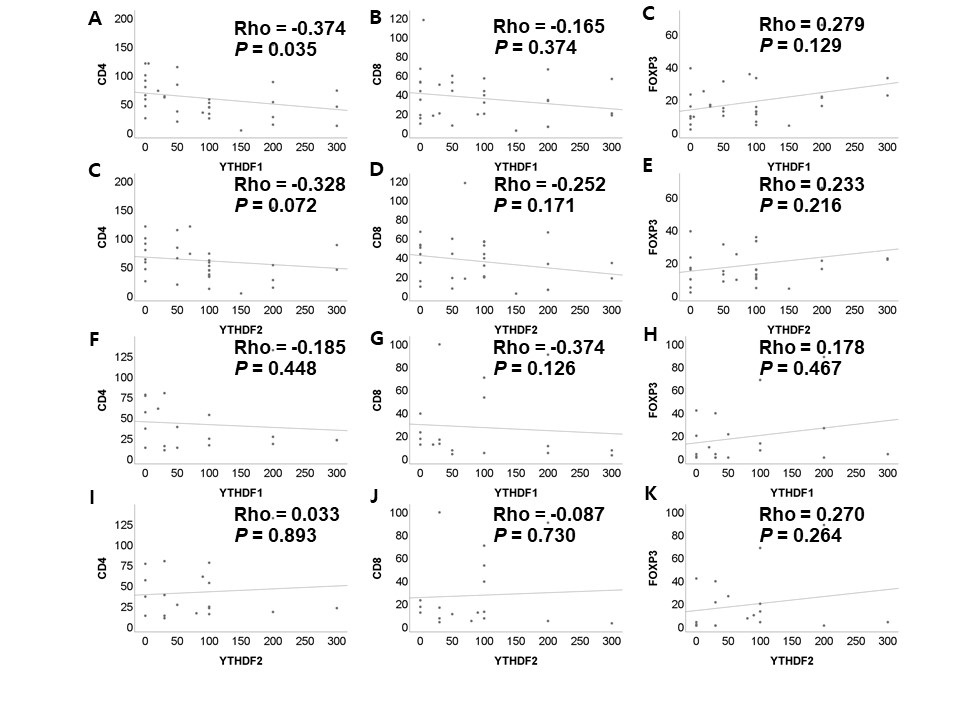

Supplement: Supplementary Figure 1 — Correlation among YTHDF1, YTHDF2, CD4, CD8, and FOXP3 analyzed by immunohistochemistry in PD-1 inhibitor treatment groups. (A) YTHDF1 and CD4 in adenocarcinoma. (B) YTHDF1 and CD8 in adenocarcinoma. (C) YTHDF1 and FOXP3 in adenocarcinoma. (D) YTHDF2 and CD4 in adenocarcinoma. (E) YTHDF2 and CD8 in adenocarcinoma. (F) YTHDF2 and FOXP3 in adenocarcinoma. (G) YTHDF1 and CD4 in squamous cell carcinoma. (H) YTHDF1 and CD8 in squamous cell carcinoma. (I) YTHDF1 and FOXP3 in squamous cell carcinoma. (J) YTHDF2 and CD4 in squamous cell carcinoma. (K) YTHDF2 and CD8 in squamous cell carcinoma. (L) YTHDF2 and FOXP3 in squamous cell carcinoma. [file Image_1.jpeg]
